# The Capstone ePortfolio in an Undergraduate Public Health Program: Accreditation, Assessment, and Audience

**DOI:** 10.3389/fpubh.2019.00125

**Published:** 2019-05-31

**Authors:** Andrew Harver, Pilar d. Zuber, Heather Bastian

**Affiliations:** ^1^Department of Public Health Sciences, University of North Carolina Charlotte, Charlotte, NC, United States; ^2^Communication Across the Curriculum, Office of Undergraduate Education, University of North Carolina Charlotte, Charlotte, NC, United States

**Keywords:** capstone, competencies, ePortfolio, high-impact practice, reflection, undergraduate public health education

## Abstract

The Bachelor of Science in Public Health (BSPH) degree program at the University of North Carolina at Charlotte (UNC Charlotte) was launched in 2007, and was initially accredited by the Council on Education for Public Health in 2009. We admit approximately 40–45 students each fall to the upper division major, through a competitive admissions process. During the junior and senior years, BSPH majors complete a core set of required courses including internship; 18 credit hours of restricted electives; and any minor offered by the university (except public health). During 2014–2015, the Department of Public Health Sciences was one of five campus units supported by UNC General Administration to pilot the use of ePortfolios as a tool to help students integrate learning across the courses that make up the major. The pilot program continued for 2 additional years, to promote enduring faculty efforts. We subsequently outline the development and implementation of ePortfolio pedagogy in the BSPH program at UNC Charlotte, including preliminary assessment of outcomes the past 3 years. The adoption of ePortfolios has been instrumental in students' educational experiences for over 2 decades. The Association of American Colleges and Universities (AAC&U) has advocated that “ePortfolios allow faculty and other educational professionals to help students organize their learning; preserve the variety of forms in which their learning occurs; and reflect upon their learning.” We have learned that effective student ePortfolios do not arise in a vacuum. In collaboration with like-minded campus colleagues including those associated with the university's Communication Across the Curriculum program, we have encountered contributing forces related to the process of “collection, selection, and reflection” including intentional assignments that yield effective student artifacts; and authentic feedback to students through adoption and modification of the AAC&U VALUE rubrics. We conclude that internal and external forces drive the development of ePortfolio content; students embrace opportunities to document learning when those opportunities are structured; the development of the ePortfolio is relational—consistent with student attributes; and ePortfolios enable evidence-based approaches to meet accreditation demands, assessment needs, and workforce expectations.

## Introduction

In 2005, the Association of American Colleges & Universities (AAC&U) launched the Liberal Education and America's Promise (LEAP) challenge to advocate the importance and relevance of a liberal education (1). The LEAP initiative is responsive to the needs of employers for more college-educated workers and more engaged and informed citizens [e.g., (2)]. At the same time, the need to increase access to higher education for a broad and diverse population has informed the necessity of more diverse pedagogies and assessments to reflect student learning. High-impact educational practices (HIPs)—pedagogies that have been shown to differentially engage and challenge students—include collaborative assignments, first year programs, intensive writing courses, internships, study abroad, and undergraduate research [3]. These teaching and learning practices have been widely vetted and have been shown to be beneficial for college students from many backgrounds, especially historically underserved students who often do not have equitable access to high-impact learning (3, 4). Since 2008, AAC&U has led the higher education community in promoting access to high-impact practices for all students and in testing the impact of HIPs on student learning outcomes. These efforts are especially relevant to non-traditional students who have different needs compared to traditional college students (5). In 2016, AAC&U added ePortfolios to the list of HIPs, as the eleventh high-impact practice (6).

Digital repositories of student work—ePortfolios—have addressed changes in students' educational experiences for over 2 decades (7, 8). The electronic or digital portfolio is ideal “for collecting evidence of student learning, especially for those outcomes not amenable nor appropriate for standardized measurement” ^1^. AAC&U has advocated that “ePortfolios allow faculty and other educational professionals to help students organize their learning; preserve the variety of forms in which their learning occurs; and reflect upon their learning”^1^. Higher education institutions are proceeding in ways that support the ePortfolio idea. For example, faculty, other educational professionals, and institutional leaders are embracing ways to meet the capacity of students to manage their own learning and to develop their own agency and identity as they follow less predictable career paths (9, 10). The peer-reviewed *International Journal of ePortfolio*; recent guides, handbooks, and other publications (8, 11–13); and release of *The Field Guide to ePortfolio*—a publication driven by the Association for Authentic, Experiential, and Evidence-Based Learning (AAEEBL) and disseminated in fall 2017 by AAC&U involving over 50 authors from the ePortfolio field (7)—attest to an ePortfolio contagion; an epidemic of sorts. Alongside these occurrences, however, calls for rigorous ePortfolio research (e.g., do ePortfolios have an enduring value to students beyond graduation; does ePortfolio use lead to higher retention and/or graduation rates?) should not be ignored (14, 15).

During 2014–2015, the Department of Public Health Sciences was one of five campus units supported by UNC General Administration to pilot the use of ePortfolios as a tool to help students integrate learning across the courses that make up the major. The pilot program continued for 2 additional years, to promote enduring faculty efforts around not only ePortfolio pedagogy but also related contours including backward design, formative feedback, intentional assignments, and scaffolding. During spring 2016, for example, about 2 dozen faculty from across campus met bi-weekly to engage in semi-structured discourse around the recent volume by Reynolds and Patton (13). Those interactions resulted in several multidisciplinary panel presentations at professional conferences (16–19).

We subsequently outline the development and implementation of ePortfolio pedagogy in the BSPH program at UNC Charlotte, including preliminary assessment of outcomes—both qualitative and quantitative—from the past 3 years.

## Background and Rationale

Fundamentally, the ePortfolio is an electronic collection of artifacts assembled over time intended to enhance student learning. We would be remiss to attempt, for the purposes of this paper, an adequate overview of ePortfolio pedagogy; we encourage instead that both the novice and expert consult the available guides and handbooks compiled by current thought leaders [e.g., (8, 10, 12, 13)]. The *Field Guide to Eportfolio* (7) in particular offers a lens—in its tone, rigor, and utility—through which to view “a burgeoning and developing field of practitioners keen to explore the potential and power of ePortfolios at their institutions.”

There may be no word used more frequently in the same sentence as “ePortfolio” than “reflection.” In nearly every related publication, reflection—“Active, persistent, and careful consideration of any belief or supposed form of knowledge in the light of the grounds that support it, and the further conclusions to which it tends” (20)—is central to ePortfolio practice and engenders the pulse of student learning [e.g., (21–23)]. At times, though, it seems that the idea of reflection is a little like fog—encompassing and manifest but lacking edges or boundaries. We do not find it surprising, therefore, that there is no stand-alone rubric for “reflection” in the current inventory of AAC&U VALUE rubrics^2^.

## BSPH Program Goals and Competencies

The BSPH program at UNC Charlotte prepares students through didactic and practice experiences to apply core principles of public health education within a variety of community settings and to advance the public health profession. The program values professional and academic integrity and ethics, collegiality, engagement with the community, and responsiveness and innovation in its pursuit of attaining the highest possible standards of health and well-being. Practical experiences (i.e., internship and capstone) are designed to reinforce the knowledge and analytical skills obtained in classroom settings including study of published research.

The curriculum is designed to prepare scholar-practitioners with knowledge and skills in the core concepts of public health, including health behavior, research and statistics in health, environmental health, epidemiology, and health administration, as well as in the planning, evaluation, organization, and conduct of community and public health services. The planned course of study adopts an interdisciplinary focus and includes the development of tailored skills through the successful completion of a minor, electives, and experiential learning (see Table 1). Our goal is to prepare students who are particularly interested in pursuing health-related careers in health promotion, program delivery, health communication, community organization, and behavior change for entry level to mid-level positions in service and research in health departments, public health agencies, community-based organizations, outreach education programs, hospitals, private health organizations, and corporate wellness settings.

**Table 1:** BSPH program core curriculum and elective requirements (credit hours).

**Required core courses (32 h)**	**Restricted electives (18 h)**
HLTH 3102 Comparative Healthcare Systems (3) HLTH 3103 Behavior Change Theories & Practice (3) HLTH 3104 Research & Statistics in Health (3) HLTH 3104L Research & Statistics in Health Lab (1) HLTH 3105 Public Health Education & Promotion (3) HLTH 4102 Healthcare Administration (3) HLTH 4103 Environmental Health (3) HLTH 4104 Epidemiology (3) HLTH 4105 Program Planning & Evaluation (3) HLTH 4105L Program Planning & Evaluation Lab (1) HLTH 4400 Public Health Internship (3) HLTH 4600 Public Health Capstone (3)	Culture & Health Courses (6) Health-Related Electives (12)

The competency-based BSPH program includes:

- instructional goals (to develop student competency in the core areas of public health; and to develop student competency to inform, assist, and promote public health through critical thinking, analysis, and synthesis of health information);
- research goals (to engage students in public health-related activities and programs in the community; and to develop oral and written communication skills to disseminate public health scholarship); and
- service goals (to encourage student involvement in public health-related local, regional, and national professional organizations; and to provide opportunities for student development as a practice professional).

The UNC Charlotte Public Health Programs received its initial (5-year) accreditation from the Council on Education for Public Health in June 2009, and subsequent re-accreditation for a 7-year period, through 2021. Like all programs that meet national standards of excellence, we are continuously engaged in the assessment, review, and improvement of our competency matrices including, in our case, those specific to the undergraduate arm of our unit of accreditation. In spring 2015, for example, the BSPH Program Committee reformulated program core competencies to devise a more manageable and ultimately streamlined set of student learning outcomes to include:

- frameworks from the social and behavioral sciences used in public health practice;
- planning, implementation, and evaluation of public health interventions;
- evidence-based approaches to evaluation and decision-making;
- public health laws, regulations, and policies integral to public health practice; and
- adaptive approaches to problems that take into account diverse communities.

### Interdisciplinary and Cross-Cutting Competencies

Faculty advocated early for an integrative approach in the development of the BSPH program content and curriculum. For example, our institutional Student Learning Outcomes pivot on the capacity of students to respond to health-related problems; to analyze and interpret the results of studies, projects and programs related to the public's health; to design an intervention using a public health model; and to communicate public health messages to diverse audiences. Student performance in the BSPH capstone course (HLTH4600)—and completion of the capstone ePortfolio in particular—is a contributing factor in the assessment and continuous improvement of student learning. We intend for the capstone ePortfolio to document carefully selected and purposeful organization of professionally related academic accomplishments that address the required competencies of the major and—for those students who are committed to a public health practice career—the major areas of responsibility and competencies for health educators. We designed our curriculum intentionally not only around the core disciplines of public health but also around a set of focused cross-cutting competencies to support our integrative approach to the development of the next generation of public health workers.

**Figure 1:**
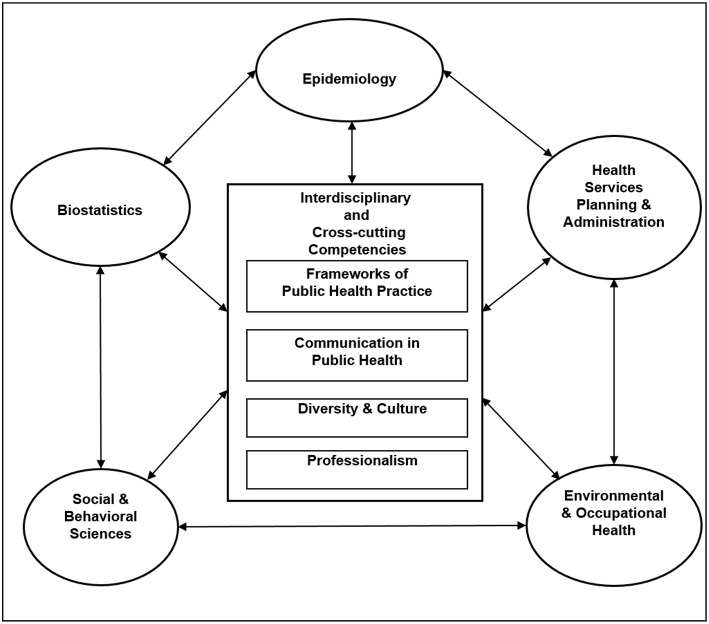
Model of the BSPH program interdisciplinary and cross-cutting competencies.

Our BSPH program cross-cutting competencies—which are included among our CEPH accreditation reporting activities—include frameworks of public health practice, communication in public health, diversity and culture, and professionalism (see Figure 1). Frameworks of public health practice address fundamentals that underlie public health practice including assessment of health status and application of the core functions of assessment, program and policy development, assurance, and communication in the analysis of public health problems and their solutions. Communication in public health emphasizes the ability to collect, manage, and organize data to produce information and meaning; and to gather, process, and present information to different audiences either in-person, through information technologies, or through media channels. Diversity and culture addresses the ability of public health professionals to differentiate among availability, acceptability, and accessibility of health care across diverse populations; and to interact sensitively, effectively, and professionally with persons from diverse cultural, socioeconomic, educational, racial, ethnic, and professional backgrounds, and persons of all ages and lifestyle preferences. Finally, professionalism entails the ability to demonstrate ethical choices, values and professional practices implicit in public health decisions; to consider the effect of choices on community stewardship, equity, social justice, and accountability; and to commit to personal and institutional development.

## Learning Environment

The University of North Carolina at Charlotte (UNC Charlotte) was founded as the Charlotte Center in 1946, and joined the statewide university system in 1965. UNC Charlotte has grown to the third largest of the 17 institutions within the University of North Carolina system with a fall 2018 enrollment of 29,710 students including 5,323 graduate students distributed among 65 programs leading to Master's degrees and 24 programs leading to Doctoral degrees. UNC Charlotte enrolls more transfer students than any other North Carolina public institution and about a third of students self-report as a racial/ethnic minority.

The Department of Public Health Sciences was founded as the Department of Health Behavior and Administration on July 1, 2002 as part of the transformed College of Health and Human Services. The new Department was conceived in response to recommendations derived from UNC Charlotte's Health Commission report completed in 2000 as well as a variety of initiatives placing emphasis on population health and health behavior research. In May 2007, the Department was renamed to Public Health Sciences to better reflect the unit's larger-scale set of current and planned research programs, degree offerings, and service activities. Faculty research programs focus on individual and population health including the prevention and management of disease across the lifespan; the health status of diverse, urban communities; and population health and health care analytics. The Department values collaboration, community engagement, diversity, health equity, innovation, professionalism, and social justice.

The Bachelor of Science in Public Health (BSPH) degree program was launched in 2007, and was initially accredited by the Council on Education for Public Health (CEPH) in 2009. We admit approximately 40–45 students each fall to the upper division major, through a competitive admissions process; each year, the entering students naturally coalesce as a “cohort” (often to include establishment of “members-only” social media sites). During the junior and senior years, BSPH majors complete a core set of required courses including internship; 18 credit hours of restricted electives; and any minor offered by the university (except public health) (see Table 1). In 2012, the unit's BSPH program was among the case studies featured in the Successful Practices Project sponsored by the Association for Prevention Teaching and Research in collaboration with the AAC&U^3^. The unit is committed to fostering a “communication enhanced curriculum” and collaborates with a renewed Communication Across the Curriculum program located in a restructured Office of Undergraduate Education^4^.

## HLTH4600 Capstone

The program capstone course—which occurs during the fall semester of the senior year—involves the development of a carefully selected and purposeful organization of professionally related academic accomplishments that addresses the required competencies of the major through the design, curation, and presentation of an integrated ePortfolio. We introduce the idea of a capstone ePortfolio in one of the core courses completed during the first semester of the program (HLTH 3105 Public Health Education & Promotion) to promote, at a minimum, the habit of artifact collection throughout the BSPH program as well as in all courses and co-curricular activities in which students participate.

### Pedagogical Format

The capstone ePortfolio is designed to reflect a carefully selected and purposeful organization of professionally related curricular and co-curricular accomplishments to validate the knowledge and skills developed not only in the BSPH program but also throughout the collegiate journey. The ePortfolio becomes a digital representation of skills and accomplishments as a public health professional, as evidenced by a compilation of selected projects and activities—files, images, graphics, videos, etc.—structured around the BSPH program's Interdisciplinary and Cross-Cutting Competencies. We have found value in providing students a common organizational structure that relates to the purposes of the ePortfolio. Accordingly, students organize their ePortfolio around the four Interdisciplinary and Cross-Cutting Competencies; each ePortfolio includes a section (or page) for each competency. In addition, students are required to design and develop one additional section (or page) of their choosing, to further tailor the representation of their growth and development as students and emerging professionals. We spend time exploring—through classroom discussion, review of previous student examples, and presentations of current works in progress—what the choice page could entail. Despite the talent evident among our BSPH majors, we find students are frequently challenged by the task of envisioning a self apart from the required components of the degree program. In the end, however, students uncover a facet of their individualism to complement their classroom education including, for example, career goals (e.g., health educator), international encounters (e.g., mission trips and/or study abroad), and personal qualities (e.g., leadership).

Students engage in reflective activities at two levels. First, they are required to begin each section (or page) with a one-to-two paragraph introduction. The introduction 1) summarizes the competency in their own words and otherwise introduces the viewer to the page; 2) describes the artifacts included in the section, and how the artifacts connect to an understanding and application of BSPH program goals; and 3) integrates the collection of artifacts as indicators of preparedness for post-graduation. Each section includes a minimum of 3–5 work samples. We remind students repeatedly “the point of the ePortfolio is to document from multiple perspectives evidence that selectively demonstrates competence to direct your own development.”

Second, the final page of the ePortfolio is limited to a final 2–3 page essay designed for students to reflect on the encompassing effects of their final, integrated product. We use the following prompts to stimulate, in part, the nature and content of the final reflection:

- What does the ePortfolio mean to you as a student, writer, or critical thinker?
- What was especially satisfying to you about either the process or final product?
- What one thing do you want people to notice when they look at your work?
- How do the various sections of the ePortfolio make sense together, and what are the larger implications or importance of your artifacts, experiences, and activities?
- Describe your learning process during the design and development of the ePortfolio.
- What were your successes and difficulties in completing the capstone ePortfolio?

### Course and Learning Objectives

The BSPH capstone course is configured around three objectives. First, students engage in the identification of personal and professional targets that inform short- and long-term career goals (facilitated, for example, through the conduct of informational interviews, mock interviews, and time management exercises). Second, students deliver a well-rehearsed presentation to demonstrate application of population health statistics from local, state, national, and global levels to inform a Healthy People 2020 Topic Area. Finally, students document knowledge and skills acquired throughout the BSPH program through the design, curation, and presentation of an integrated ePortfolio.

The BSPH capstone course not only contributes to CEPH-related criteria (i.e., health communication: address the basic concepts of public health-specific communication, including technical and professional writing and the use of mass media and electronic technology) but also to institutional Student Learning Outcomes (i.e., students will be able to respond to health-related problems). Accordingly, the BSPH program coordinator is involved annually in the review and reporting of course objectives and outcomes.

### Rubric and Criteria

The assessment of ePortfolios is not without challenge [e.g., (24–26)]. One approach involves the adoption of the Valid Assessment of Learning in Undergraduate Education (VALUE) rubrics advanced by AAC&U (27, 28). During 2007–2009, teams of faculty and other educational professionals from institutions across the country −2- and 4-year, private and public, research and liberal arts, large and small—developed rubrics for 16 student learning outcomes that all students need for success in work, citizenship, and life (e.g., information literacy, oral communication, quantitative literacy, teamwork, and written communication). Like at many institutions, the integrative learning VALUE rubric served as a starting point for our assessment of student learning evident in ePortfolios. Our current rubric turns on evaluation of six criteria culled and synthesized from a variety of resources (12, 13, 27–29) including artifact selection, connections to curricular and co-curricular activities, digital and visual literacy, effective communication, sense of purpose, and overall effect. We judge each criteria against the rubric, as any instructor would judge any student paper, presentation, or project. We expect as per our SLO that “90% of the students will score 80% or above on the Capstone Portfolio.”

We expect that the selection of artifacts provides strong support for the claims made in the introduction to each page as well as in the final reflective analysis about student learning processes. In addition, the text associated with each individual artifact should demonstrate both technical and professional proficiencies. We have observed that the curation of artifacts is not always an easy task for students, who seem on occasion to haphazardly distribute artifacts among the requisite pages to meet the expected minimum number of work samples. We encourage students to think through as an illustration of process the relevance of the internship report, which many students include as part of the ePortfolio. We ask students to reflect, for example, if the content of the internship report best serves as a summary of the implementation of a program (frameworks), a polished piece of writing (communication), an activity involving a vulnerable population (diversity and culture), or a summary of workforce development (professionalism). In past years—especially when we were working with paper-and-pencil compilations—students also sometimes struggled uncovering or recovering a critical mass of relevant artifacts (laptops had crashed; thumb drives had been lost; etc.). More recently, however, the “technology” has come into play to maximize the availability and recovery of selected material. For example, student assignments once uploaded through our learning management system (Canvas) remain available to students throughout their matriculation at the university. In addition—at least for group assignments and to some extent individual work as well—students have become adept using a variety of university-approved platforms such as Google Drive and Dropbox to archive papers, presentations, and projects during their time at UNC Charlotte.

Students readily make connections between the BSPH program goals and related curricular and co-curricular activities. Many of our students, for example, hold leadership positions in student organizations, serve as campus ambassadors, or complete study abroad programs and/or mission trips. All of our students complete a minor (or second major); and nearly all of our students work on and/or off campus at least part-time and we emphasize the value and relevance of transferable skills (e.g., assuming responsibility, customer service, interpersonal skills, interpreting policy, and negotiating).

We value as part of the building process the design, layout, and navigation of student ePortfolios to facilitate the development of digital and visual literacy; and we expect that all elements of the ePortfolio demonstrate careful editing and proofreading. It is always surprising to us that despite familiarity with social media (e.g., Facebook) the construction of a well-designed and clearly organized ePortfolio is far from intuitive for most students (even for those with a minor in graphic design). Although we align with many others who appraise the technology as secondary, some platforms afford an extensive array of design and navigational choices. We have been fortunate the past several years to implement through collaborative efforts educational software from Digication, a platform adopted by many institutions.

Because student ePortfolios are comprised mostly of text, evidence of effective communication is paramount to include organization, structure, clarity, and style. Relatedly, fully developed ePortfolios convey a sense of purpose to the reader; the writer is able to signal a strong sense of what she is trying to do or say. Our final criteria—overall effect—refers to the gestalt of the product. We confront the extent to which the entire ePortfolio is cohesive, convincing, and makes a strong case for its author's abilities, knowledge, and learning development.

## Preliminary Observations

**Figure 2:**
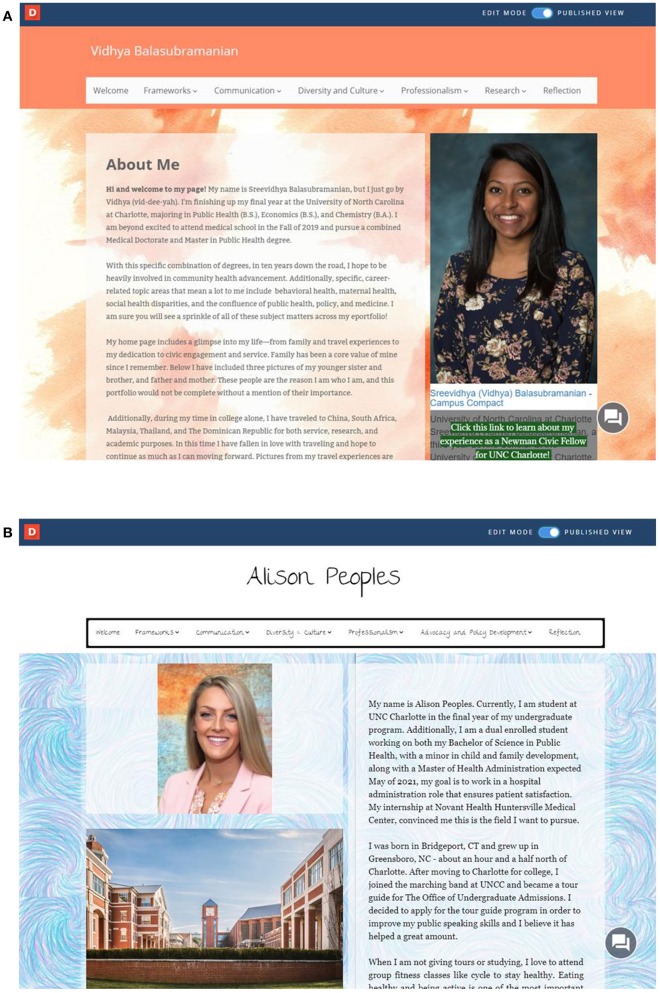
Homepages for two BSPH student ePortfolios. **(A)** Homepage for Vidhya. Used with written informed consent of the student. **(B)** Homepage for Alison. Used with written informed consent of the student.

In Figure 2, we provide screenshots of homepages for two recent BSPH student ePortfolios, from fall 2018. As is evident, in addition to the initial “welcome” or “about me” page, the top banner of our common template enables links to six additional pages. The first four are restricted to the program's four cross-cutting competencies—frameworks, communication, diversity and culture, and professionalism. As described, students also build a fifth page of their choosing, to tailor further their ePortfolio content, and are encouraged to select a theme or thread consistent with their experiences both inside and outside the BSPH program that have shaped their current goals and future aspirations. Vidhya, an aspiring MD/MPH candidate, elected to emphasize her undergraduate research experiences; Alison, an early-entry MHA student, elected to emphasize her career interests in child advocacy and policy development. The final page—reflection—serves mainly as a placeholder for students to upload the final paper at the end of the semester focused on the process of building the ePortfolio including the curation of artifacts, their organization, and their connections. Students are also required to characterize, in the final reflection, the ways in which their artifacts represent understanding and application of BSPH program goals, meet cross-cutting competency expectations, and demonstrate preparedness for post-graduation.

In each of the past 3 years we have distributed as part of the course a brief survey at the very end of the semester to collect, anonymously and voluntarily (94 of 130 total students; 72%), student ratings of outcomes (one of us—AH—was the instructor of record on all three occasions). Students rate seven questions on a five-point scale (either ranging from “strongly disagree” to “strongly agree,” or ranging from “not at all” to “very much”). We summarize our findings from these preliminary efforts to assess ePortfolio impact in Table 2. Although relatively uniform, we attribute variability in the ratings evident during 2017–2018, for example, in part to the transition to a new ePortfolio platform (like faculty, students are oftentimes frustrated with new technology). We derived our survey questions based on the Connect to Learning (C2L) Core Survey, collected from nearly 10,000 students across the campuses involved in exploring and documenting ePortfolio strategies to advance student learning (8, 30). We include for comparative purposes weighted averages from the past 3 years with equivalent values obtained from among the 24 campuses with established ePortfolio projects that participated in the C2L project [(30); Table 2].

**Table 2:** Student ratings of BSPH ePortfolio outcomes.

**Outcomes**	**Academic year**	**2016-17 (*n* = 34)**	**2017-18 (*n* = 27)**	**2018-19 (*n* = 33)**	**Weighted average (*n* = 94)**	Equivalent values from the C2L core survey^***^ (*n* = 9,542)
^*^Building my ePortfolio…	helped me make connections between ideas and experiences	88%	78%	89%	85%	70%
	helped me think more deeply about the BSPH program content	79%	78%	79%	78%	62%
	allowed me to become aware of my development as a learner	77%	63%	89%	77%	65.6%
	prepared me to think more fully about my career readiness	88%	78%	89%	85%	70%
^**^My ePortfolio course engaged me in…	synthesizing & organizing ideas, information, or experiences in new ways	88%	52%	68%	70%	78.3%
	applying theories or concepts to practical problems or in new situations	76%	41%	52%	57%	73.6%
	advancing my knowledge, skills, and personal development	94%	70%	78%	81%	74.1%

* Proportion of students who rated the item as “agree” or “strongly agree.”

** Proportion of students who rated the item as “quite a bit” or “very much.”

*** *Eynon et al. (30)*.

As described earlier, students begin each section (or page) with a one-to-two paragraph introduction that summarizes the competency in their own words, describes the artifacts included in the section, and integrates the collection of artifacts as indicators of preparedness for post-graduation. In Table 3, we provide excerpts from a sample of student reflections extracted from the introduction to one cross-cutting competency (diversity and culture). We judge that there is an element of consistency among these excerpts aligned with our expectations. They demonstrate, for example, application of public health principles (e.g., “I will know how to…differentiate among availability, acceptability, and accessibility of health care across diverse populations;” “In order to be prosperous in public health, a person must be able to demonstrate ethical choices for the greater good of the community and to ensure social justice”). They also meet competency expectations (e.g., “Diversity is a valuable asset to any organization, but it has a unique importance in public health. A diverse workforce will be better equipped to deal with public health issues that may arise in communities”) and address workforce readiness (e.g., “These experiences improved my cultural competency, cross-cultural communication, ability to navigate ambiguous situations, and creativity regarding health communication materials”).

**Table 3:** Sample student reflections: culture and diversity.

Public health professionals must appreciate working alongside different organizations and agencies within the community and be able to use them as resources and for knowledge. It is important to interact with individuals with integrity and incorporate a shared understanding of the goals set in place to affect the outcome of a health issue. It is also essential to understand the availability of resources within different communities, the accessibility to health services, and the willingness of communities to take advantage of those resources in understanding how to approach working with different audiences.
Diversity is a valuable asset to any organization, but it has a unique importance in public health. A diverse workforce will be better equipped to deal with public health issues that may arise in communities. Diversity allows for the inclusion of populations often marginalized. Humans tend to be more receptive to individuals with similar cultural characteristics. The importance of diversity in public health is evident through the selection of the students in the BSPH program. BSPH students vary widely in ethnicity, age, and professional experience. We work closely with individuals within our cohort in order to foster relationships with diverse populations.
The support of the BSPH program at UNC Charlotte allowed me to take a semester abroad in Barcelona, Spain examining their public health system, followed by a summer internship in Cape Town, South Africa being directly involved with South African healthcare delivery. These experiences improved my cultural competency, cross-cultural communication, ability to navigate ambiguous situations, and creativity regarding health communication materials. My BSPH experiences only reconfirmed for me that I want to continue working with diverse and vulnerable populations.
The competencies work together to create a holistic view of a public health professional. A successful health professional encompasses strengths in each competency to produce the highest possible standard of health and well-being…Public health impacts everyone, and is not limited to one population; therefore, the ability to work with diverse populations, along with the ability to be sensitive to cultural differences, is required. In order to be prosperous in public health, a person must be able to demonstrate ethical choices for the greater good of the community and to ensure social justice. Overall, the application of each of these competencies work together to produce the outcome, which is well-being in all dimensions.
Diversity and culture address the ability of public health professionals to interact with diverse individuals and communities, with integrity and shared values, to produce or affect an intended public health outcome. As a BSPH graduate, I will know how to develop and adapt approaches to problems that take into account cultural differences and identify community assets and available resources, differentiate among availability, acceptability, and accessibility of health care across diverse populations, and appreciate the importance of working collaboratively with diverse communities and constituencies.

## Discussion

Although about half of all colleges report some use of ePortfolios (8), <2 dozen published papers among the over 500 ePortfolio peer-reviewed journal articles part of the PEARL (Publications on ePortfolio: Archives of the Research Landscape)^5^ database focus on “capstone.” Our report is the first to provide an overview of the development, implementation, and (preliminary) assessment of ePortfolios in a CEPH-accredited undergraduate public health capstone course. Our cumulative experiences to date—albeit within the confines of a tightly knit upper division program—compare favorably with the respected impact of the national C2L effort (8). An overwhelming proportion of undergraduate students reported particular value documenting acquired knowledge and skills through the design, curation, and presentation of an integrated capstone ePortfolio. The ePortfolio process contributed to making connections between ideas and experiences, and to thinking more deeply about the BSPH program content; and engagement in the activity was perceived relevant to personal development and to career readiness.

The purpose of ePortfolios is often multi-faceted and relevant to students, agencies and institutions, and external stakeholders. There is of yet no master agreement on the key value of “ePortfolio”—both process and product is inherently adaptable, elastic, and inventive. On the other hand, few would disagree with the outline by AAC&U that the majority of work around the adoption of ePortfolios relates to student learning, institutional assessment, and professional development and employment^6^. We briefly address the implicit and explicit elements of these varied narratives in terms of the applicability of ePortfolios to meeting professional standards (accreditation), monitoring student learning (assessment), and cultivating student identities relevant to external stakeholders (audience).

### Accreditation

We have found that disciplinary frameworks, program goals, and a competency-based curriculum provide a latent organizational structure for the design and layout of an ePortfolio. In this way, students selectively populate content to showcase instances of learning aligned with programmatic instructional, research, and service goals as well as articulate with clarity connections evident among course assignments, practical experiences, co-curricular activities, and transferable skills through reflective practice. Accredited degree programs of all types assure a continuous cycle of quality improvement involving students, faculty, administration, and community stakeholders. In previous years, CEPH site team members have had interest in reviewing selected paper-and-pencil portfolios. We look forward to future judgments of the perceived utility of ePortfolios to convey the proficiency of entry-level professional personnel who are able to identify, prevent, and solve community health problems.

### Assessment

Few would argue that recent alarms over the access, cost, and quality of higher education—both imagined and real—have intensified institutional learning-outcomes assessment efforts. On the other hand, there is an increased authenticity associated with integrative assessment practices on most campuses to provide meaningful evidence of student learning that guide institutional decision-making to improve student performance. Monitoring and responding to student performance levels, however, are not new activities for professional degree programs such as public health. Student learning outcomes (e.g., students will be able to respond to health-related problems) are corroborated when assignments and rubrics are mapped to program competencies and shared for review and reflection among participating faculty.

### Audience

We have emphasized the potential “outward facing” value of ePortfolios, especially in terms of workforce readiness. Such an emphasis is consistent with results of a recent survey involving 400 employers whose organizations have at least 25 employees and report that 25% or more of their new hires hold either an associate degree from a 2-year college or a bachelor's degree from a 4-year college. Most employers (80%) say they would find an electronic portfolio of a candidate's work very (36%) or fairly (44%) useful to evaluate key skill and knowledge areas (2). We are learning that our students often now add the URL for their ePortfolio in job applications. We will be curious to track where ePortfolios might fall in the future along a line of enriched digital presences that might include, for example, LinkedIn and enhanced transcripts (31).

The ePortfolio entails an intrinsic “inward facing” value as well. The process of curating artifacts and content requires students to make organizational decisions not encountered preparing for tests or completing a 10-page research paper (32). Students often experience a new kind of discomfort when they are confronted to both discover and uncover, in a way, a mirror image (a reflection?), and the *raison d'être* for the ePortfolio. On the other hand, the current generation of traditional students—and we accept that they may not represent a majority of the student body at many institutions—value interactive, relevant, and technology-based learning environments (33).

### Final Thoughts

Over 20 years ago faculty throughout higher education were pressed with a rising expectation—which never gained lasting traction—that the development of effective communication skills in students, especially writing, was the responsibility of every member of the academic community. Higher education institutions are now proceeding in ways that support the ePortfolio idea, and authentic, student-centered learning. Such momentum, however, inadvertently adds to the mix of new technologies—learning management systems, degree progress verifications, and interactive learning products—that faculty are expected to master to meet new efficiencies, expedite reporting, and confront student attention spans. Although the technology that puts the ePortfolio idea into practice is secondary, the exploration and ultimate implementation of an ePortfolio course—or curriculum—competes with an ever-increasing array of advising, coaching, community engagement, mentoring, and service expectations.

We have learned that effective student ePortfolios need not arise in a vacuum, and flourish when collaborative faculty both inside and outside an academic unit establish and reinforce instructional expectations, both formally and informally. We encourage others to coordinate ePortfolio efforts with other campus initiatives and priorities to help mitigate the inevitable fatigue that accompanies early adoption of an innovation. It is important to note, however, that such endeavors—time intense, oftentimes risky, and frequently fraught with failure—are not always part of routine faculty annual evaluations, which can pose a significant challenge to the implementation and sustainability of grassroots ePortfolio initiatives. Higher levels of both faculty and student satisfaction, on the other hand, may offset the burden that often accompanies evaluation of novel pedagogical practices.

Technology choices are influenced by both “top-down” and “bottom-up” decision processes (7) and further complicate implementation and sustainability factors. As campuses scale the adoption of ePortfolios tensions can arise, as a natural sequela of complex organizations, around who fundamentally best manages ePortfolio processes or contributes most to support services and faculty development: possible players include campus equivalents of the Center for Teaching and Learning, Information Technology Services, Office of Assessment, and Writing or Communication Across the Curriculum Programs. Batson, for example, recently lamented (34):

“Institutions realized by 2003 or 2004 that student work stored on the web could be easily “mined” in aggregate for institutional assessment. In an instant, electronic portfolios became the “magic bullet” for institutional assessment…Nothing is wrong with that use, of course, but, as I wrote at the time, the eportfolio learning idea had been, so to speak, “hijacked” by institutional assessment needs; requiring professors to use eportfolios for institutional purposes was not the way to nurture a precious learning idea.”

Despite a variety of prevailing challenges we concur, and close, with Batson's call to action (34):

“Higher education needs to lead the world, and leaders in higher education have to see that larger mission to understand what their institution must do to change. Eportfolio practice is one way to grasp the kinds of changes the entire institution must work toward.”

## Conclusion

We conclude that internal and external forces (e.g., institutional assessment needs and professional accreditation expectations) can drive the development of effective ePortfolio purpose and content, and that students embrace opportunities to document learning when those opportunities are structured around programmatic goals. The multi-faceted value of ePortfolios enables evidence-based approaches suitable for addressing accreditation expectations, meeting institutional assessment needs, and documenting preparedness for contributions to the workforce. Accreditation criteria and related curriculum efforts serve as ready-made organizing principles. Institutional Student Learning Outcomes reinforce programmatic goals. External stakeholders value authentic presentation of acquired skills. Unlike some high impact practices that have been shown to differentially engage and challenge students (e.g., internships and study abroad), ePortfolios are within reach of every student. The development of the ePortfolio is relational—akin to the development of creating patterns and making connections among objects and people—and, accordingly, appealing to a large portion of the current student body at institutions of higher learning. In short, we encourage the adoption of ePortfolios throughout undergraduate public health degree programs to widen the reach of effective high-impact educational practices and inclusive learning opportunities.

## Data Availability

All datasets analyzed for this study are included in the manuscript.

## Ethics Statement

Our study was reviewed by the Office of Research Compliance at UNC Charlotte (#19-0090) and was determined to meet the Exempt category (1. Educational setting) under 45 CFR 46.101(b).

## Author Contributions

All authors listed have made a substantial, direct and intellectual contribution to the work, and approved it for publication.

### Conflict of Interest Statement

The authors declare that the research was conducted in the absence of any commercial or financial relationships that could be interpreted as a potential conflict of interest.

## Footnotes

^1^https://www.aacu.org/eportfolios.

^2^https://www.aacu.org/value-rubrics.

^3^https://www.aptrweb.org/page/SuccessfulPractices.

^4^https://cxc.uncc.edu/.

^5^https://eportfolio.aacu.org/

^6^https://www.aacu.org/eportfolios.
